# An untargeted metabolomics strategy to measure differences in metabolite uptake and excretion by mammalian cell lines

**DOI:** 10.1007/s11306-020-01725-8

**Published:** 2020-10-07

**Authors:** Marina Wright Muelas, Ivayla Roberts, Farah Mughal, Steve O’Hagan, Philip J. Day, Douglas B. Kell

**Affiliations:** 1 Department of Biochemistry and Systems Biology, Institute of Systems, Molecular and Integrative Biology, University of Liverpool, Liverpool, UK; 2 School of Chemistry, The Manchester Institute of Biotechnology, 131, Princess St, Manchester, M1 7DN UK; 3 The Manchester Institute of Biotechnology, 131, Princess St, Manchester, M1 7DN UK; 4 Faculty of Biology, Medicine and Health, The University of Manchester, Manchester, M13 9PL UK; 5 Novo Nordisk Foundation Centre for Biosustainability, Technical University of Denmark, Building 220, Chemitorvet, Kgs Lyngby, 2000 Denmark

**Keywords:** Human serum, Untargeted metabolomics, Transporters, LC-MS/MS, Orbitrap, Cell culture

## Abstract

**Introduction:**

It is widely but erroneously believed that drugs get into cells by passing through the phospholipid bilayer portion of the plasma and other membranes. Much evidence shows, however, that this is not the case, and that drugs cross biomembranes by hitchhiking on transporters for other natural molecules to which these drugs are structurally similar. Untargeted metabolomics can provide a method for determining the differential uptake of such metabolites.

**Objectives:**

Blood serum contains many thousands of molecules and provides a convenient source of biologically relevant metabolites. Our objective was to detect and identify metabolites present in serum, but to also establish a method capable of measure their uptake and secretion by different cell lines.

**Methods:**

We develop an untargeted LC-MS/MS method to detect a broad range of compounds present in human serum. We apply this to the analysis of the time course of the uptake and secretion of metabolites in serum by several human cell lines, by analysing changes in the serum that represents the extracellular phase (the ‘exometabolome’ or metabolic footprint).

**Results:**

Our method measures some 4000–5000 metabolic features in both positive and negative electrospray ionisation modes. We show that the metabolic footprints of different cell lines differ greatly from each other.

**Conclusion:**

Our new, 15-min untargeted metabolome method allows for the robust and convenient measurement of differences in the uptake of serum compounds by cell lines following incubation in serum. This will enable future research to study these differences in multiple cell lines that will relate this to transporter expression, thereby advancing our knowledge of transporter substrates, both natural and xenobiotic compounds.

**Electronic supplementary material:**

The online version of this article (10.1007/s11306-020-01725-8) contains supplementary material, which is available to authorized users.

## Introduction

One of the great unsolved problems of modern biology concerns the substrates of membrane transporters (Cesar-Razquin et al. 2018; Cesar-Razquin et al. 2015; Girardi et al. 2020; Superti-Furga et al. 2020), many of which remain ‘orphans’ (i.e. with unknown substrates), often despite the passage of decades since their identification in systematic genome-sequencing programmes (Ghatak et al. 2019).

‘Untargeted metabolomics’ is a term nowadays commonly used to describe methods that seek the reproducible detection (and sometimes quantification) of small molecules in biological matrices (Cho et al. 2014; Dunn et al. 2013; Garg et al. 2015; Martin et al. 2015; Tautenhahn et al. 2012; Treutler et al. 2016). It is most commonly performed using chromatography coupled to mass spectrometry (e.g. (Dunn et al. 2011; Dunn et al. 2007; Dunn et al. 2012)). A variety of methods, summarised in Table 1, have been developed for measuring the human serum metabolome, with both low- and high-resolution mass spectrometry. The studies highlighted in Table 1 are those we found where annotation levels were specified, and similar LC was performed. An ideal method would be rapid, reliable, and provide data on many (1000s of) metabolites simultaneously.

In a ground-breaking study using low-resolution LC-MS, Gründemann and colleagues (Gründemann et al. 2005) recognised that incubation of cells containing different levels of a transporter of interest with plasma (as an reasonably unbiased source of candidate metabolites) might allow the discovery of transporter substrates by assessing their differential uptake into cells. They thereby discovered the transporter for the important nutraceutical (Borodina et al. 2020) ergothioneine (Gründemann 2012; Gründemann et al. 2005).

The uptake and excretion of nutrients and natural products by cells is mediated by transporter proteins of two general types: solute carriers (or SLCs) and ATP-binding cassette (ABC), transporters. Around 500 known SLC transporters are known which mediate the uptake of such compounds whereas ABC transporters are involved in efflux of these(Hediger et al. 2004). Much evidence shows that drugs hitchhike on transporters for other natural molecules for which these drugs are structurally similar (Cesar-Razquin et al. 2018; Kell 2020; Kell and Oliver 2014; O’Hagan and Kell 2017; Superti-Furga et al. 2020). Moreover, recent work by Girardi et al. highlighted the key role of SLC transporters in resistance to 60 cytotoxic compounds representative of the chemical space covered by approved drugs (Girardi et al. 2020) .

As part of a wide-ranging study into the nature of transporter substrates (e.g. (Dobson and Kell 2008; Jindal et al. 2019; Kell et al. 2013; Kell et al. 2011; Kell and Oliver 2014; Kell et al. 2018)), we recognised that the approach of Gründemann and colleagues (Gründemann et al. 2005) could be an ideal strategy for implementation using modern, high-resolution metabolomics methods. We have previously developed low-resolution methods for determining the human serum metabolome reliably (Broadhurst and Kell 2006) and over extended periods (Begley et al. 2009; Dunn et al. 2011; Kenny et al. 2010; Zelena et al. 2009), including the extensive use of QA/QC samples. Our first requirement was thus to develop a new and robust method for untargeted serum metabolomics using modern, high-resolution instrumentation. The present paper describes this method, and an initial application to a series of human cell lines.

**Table 1:** Selection of untargeted LC–MS studies of human serum

References	Chromatography details	ESI–MS and polarities	MS/MS	Metabolic Features (after correction/exclusion)		MSI Identification and Annotation levels (Sumner et al. 2007)								Libraries used for identification and annotation
						Level 1		Level 2		Level 3		Level 4		
				ESI+	ESI−	ESI+	ESI−	ESI+	ESI−	ESI+	ESI−	ESI+	ESI−	
(Jiang et al. 2013)	UPLC C_18_ column Gradient time: 10 min A = water, B = acetonitrile (0.1% formic acid in A &B for ESI−)	Waters Q-TOF premier (resolution not stated) ESI+ and ESI−	Not stated	1371	6169	175		–	–	–	–	–	–	NIST 11 Standard Mass Spectral databases in NIST MS search 2.0 and in-house reference compounds (~ 800 mammalian metabolite standards)
(Dunn et al. 2015)	UPLC C_18_ column Gradient time: 22 min ESI+ , 24 ESI− , A = water + 0.1% formic acid, B = methanol + 0.1% formic acid	Waters LCT (resolution not stated) ESI+ and ESI−	No	2178	2280	–	–	659	386	–	–	1519	1894	Revised version of the Manchester Metabolomics Database (MMD)(Brown et al. 2009)
(Ganna et al. 2015)	UPLC C_18_ column Gradient time: 23 min A = 95% water, 5% methanol, 0.1% formic acid, B = 95% methanol, 5% water, 0.1% formic acid	Waters Xevo G2 QTOF MS (resolution not stated) ESI+	Indiscriminant MS/MS (also called MS^E^)	9753/10,160/7520*	–	109	–	102	–	18	–	–	–	In house spectral library or using publicly available databases

Note that only studies found where metabolic features as well as descriptions of metabolite identification levels have been included. MSI Identification levels: Level 1, proposed structure confirmed via measurement of reference standard with MS, MS/MS and retention time matching, along with, ideally, an orthogonal method; Level 2: putative annotation based on matching based on physicochemical properties and or spectral similarity with spectral libraries, Level 3: putatively characterised compound class based upon match to mass or spectral library, or experimental data; Level 4: unknown compound

*This study performed analyses with 3 subsets of samples

## Methods

### Cell culture

A549, K562, SAOS2 and U2OS cell lines were cultured in RPMI-1640 (Sigma, Cat No. R7509) culture media supplemented with 10% fetal bovine serum (Sigma, Cat No. f4135) and 2 mM glutamine (Sigma, Cat No. G7513) without antibiotics. Cell cultures were maintained in T225 culture flasks (Star lab, CytoOne Cat No. CC7682-4225) kept in a 5% CO_2_ incubator at 37 °C until 70–80% confluent. The A549 cell line was purchased from the European Collection of Authenticated Cell Cultures (ECACC, Salisbury, UK), K562 cell line was a kind gift from Dr Philip J. Day (The Manchester Institute of Biotechnology, The University of Manchester), SAOS2 and U2OS cell lines were a kind gift from Prof. Peter Gardner (The Manchester Institute of Biotechnology, The University of Manchester).

### Serum incubation experiments

#### Harvesting cells for serum incubation experiments

Cells from adherent cell lines were harvested by removing growth media and washing twice with 5 mL of pre-warmed Dulbecco’s Phosphate Buffered Saline (PBS) without calcium or magnesium (Gibco, Cat No. 14190094), then incubated in 3 mL of Gibco™ TrypLE™ Express Enzyme (1X), no phenol red (Gibco Cat No. 12604013) for 2–5 min at 37 °C. At the end of incubation cells were resuspended in 5–7 mL of respective media when cells appeared detached to dilute TrypLE treatment. The cell suspension was transferred to 50 mL centrifuge tubes and immediately centrifuged at 300 × g for 5 min. Suspended cell lines were centrifuged directly from cultures in 50 mL centrifuge tubes and washed with PBS as above. The cell pellets were resuspended in 10–15 mL media and cell count and viability was determined using a Countess II FL Automated Cell Counter (ThermoFisher Scientific) set for Trypan Blue membrane exclusion method. Cells with > 95% viability were used for serum incubation experiments.

#### Incubation of cells in serum

**Fig. 1:**
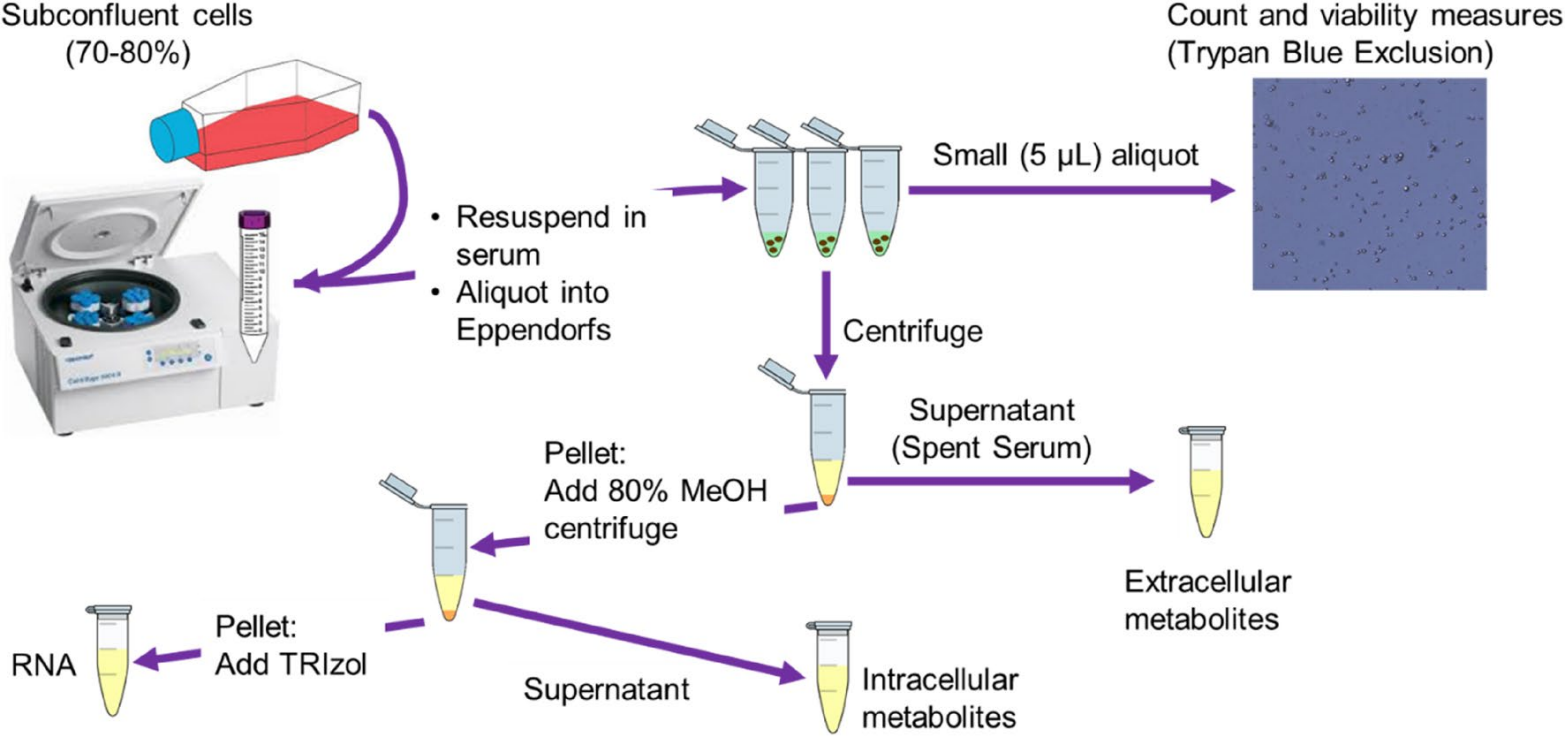
Incubation of cells in serum for metabolomics analysis to determine transporter substrates. Following incubation of cells in serum, spent serum is collected after centrifugation, followed by extraction using methanol. The remaining cell pellet is washed with PBS (at 37 °C), followed by quenching and extraction of intracellular metabolites using 80% methanol. The spent medium and intracellular extracts are subsequently lyophilised (with a mixture of internal standards spiked in prior to lyophilisation) and reconstituted in water ready for analysis by LC-HRMS/MS

The procedure for incubation of cells in serum is described pictorially in Fig. 1. More detailed information is provided in Supplementary Information file.

### Internal standard solution mixture

An internal standard stock mixture was prepared using the following compounds and concentrations: citric acid-d_4_ (Cambridge Isotope Laboratories, DLM-3487), 225 µM; L-lysine-d_4_ (Cambridge Isotope Laboratories, DLM-2640), 112.5 µM; L-Tryptophan-(indole-d_5_) (Cambridge Isotope Laboratories, DLM-1092), 5.625 µM; stearic acid-d_35_ (Cambridge Isotope Laboratories, DLM-379), 225 µM; succinic acid-d_4_ (Sigma 293,075), 112.5 µM; ^13^C_6_-carbamazepine (Sigma, C-136), 2.25 µM; Leucine-d_10_ (Sigma 492,949), 22.5 µM and methionine-d_4_ (Cambridge Isotope Laboratories, DLM-2933), 22.5 µM.

### Sample preparation for metabolomics analysis

Fresh or spent serum samples were thawed at room temperature and maintained on ice throughout the sample preparation process. Samples were prepared by addition of 100 µL sample to a 2 mL Eppendorf containing 330 µL Methanol (LC-MS grade) and 20 µL of internal standards mix (ISTDs). The mixture of methanol and ISTDs was previously cooled at − 80 °C and maintained on dry ice when adding serum. The mixture of serum, methanol and ISTD was vortexed vigorously followed by centrifugation at 13,300 rpm for 15 min at 4 °C to pellet proteins. Multiple 75 µL aliquots (for extraction replicates) of the resulting supernatant dried in a vacuum centrifuge (ScanVac MaxiVac Beta Vacuum Concentrator system, LaboGene ApS, Denmark) with no temperature application and stored at − 80 °C until required for LC-MS/MS analysis.

Quality controls (QC) and conditioning QC samples were also prepared in this way using pooled human serum.

Extraction blanks were prepared in the same way as spent serum samples replacing serum and internal standard mix with 120 µL of water (LC-MS grade). Evaluation/system suitability samples were also prepared by replacing serum with water.

Prior to analysis, samples were resuspended in 40 µL water (LC-MS), centrifuged at 13,300 rpm for 15 min at 4 °C to remove any particulates and transferred to glass sample vials.

### HPLC-MS /MS analysis of spent serum samples

Untargeted HPLC-MS/MS data acquisition was performed following methodologies and guidelines in (Broadhurst et al. 2018; Broadhurst and Kell 2006; Brown et al. 2005 ; Dunn et al. 2011 ; Mullard et al. 2015 ). Data were acquired using a ThermoFisher Scientific Vanquish HPLC system coupled to a ThermoFisher Scientific Q-Exactive mass spectrometer (ThermoFisher Scientific, UK). A resolution of 70,000 was used for MS and 17,500 for ddMS (further details provided in Supplementary Information). Chromatographic separation was performed on a Hypersil Gold aQ column (C18 2.1 mm × 100 mm, 1.9 µm, ThermoFisher Scientific) operating at a column temperature of 50 °C. Elution was performed over 15 minutes at a flow rate of 0.4 mL/min using two solvents: 0.1% formic acid in water (solvent A) and 0.1% formic acid in methanol (solvent B). as described in Table 2 below. Column eluent was diverted to waste in the first 0.4 min and the last 0.1 min of the gradient. Sample vials were stored at 4 °C in the HPLC autosampler, with 5 µL injected for positive ionisation and 15 µL for negative ionisation. MS acquisition settings are described in supplementary information 1.

**Table 2:** HPLC gradient elution program applied for HPLC-MS/MS analysis for ESI+ and ESI− modes

Time (minutes)	Flow rate (mL/min)	Solvent A (%)	Solvent B (%)
0	0.4	99.0	1.0
2	0.4	99.0	1.0
10	0.4	1.0	99.0
12	0.4	1.0	99.0
13	0.4	99.0	1.0
15	0.4	99.0	1.0

Samples were analysed following guidelines set out in (Dunn et al. 2011) and (Broadhurst et al. 2018). Briefly, blank extraction samples were injected at the beginning and end of each batch to assess carry over and lack of contamination. Isotopically labelled internal standards were added to analytical and QC samples to assess system stability throughout the batch. QC samples, prepared with a standard reference material, pooled human serum, were also applied to condition the analytical platform, enable reproducibility measurements and to correct for systematic errors.

### LC-MS/MS data preprocessing and analysis

Raw instrument data (.RAW) were exported to Compound Discoverer 3.1 (CD3.1) for deconvolution, alignment, and annotation (full workflow and settings are provided in supplementary information 2). Peak areas from CD3.1 were subsequently exported as a .csv file and QC-based LOESS signal correction performed in R (version 4.0.2) as discussed in (Dunn et al. 2011) using the loess.as function of the fANCOVA package. Strict QA criteria (minimum QC coverage of 80% and maximum QC CV 30%) was applied to the resulting data.

For analysis of data for serum compound uptake and excretion by cell lines, normalised peak areas were exported as an excel file into a KNIME workflow developed in-house for data analysis and visualisation (available on request). Within this workflow, Principal Components Analysis was used to visualise trends. Subsequently, simple univariate statistical analyses were carried out on log_2_ transformed data using a paired t-test. Volcano plots were created using these data, with a threshold of P < 0.05 and absolute log_2_ fold change > 0.5 set for defining a notable change in compound abundance between time points compared. UpSet plots (Lex et al. 2014) (Supplementary Fig. 3) were then used to find unique and shared consumed or secreted compounds between cell lines.

For all data acquired, annotation and identification criteria were according to (Schymanski et al. 2014).

## Results

### A RP-LC-ESI-MS/MS method capable of detecting a broad range of serum compounds

The optimisation of chromatographic (O’Hagan et al. 2005) and mass spectrometric (Vaidyanathan et al. 2003) methods typically requires trade-offs between multiple objectives. During the development of the LC-MS/MS method used here our aim was both to maximise the number of serum compounds detected but also to acquire sufficient fragmentation data for more confident identification, with both achieved within a reasonably short period. Table 3 shows a summary of the results obtained from CD3.1 using the data acquired using the LC-MS/MS method described in both ESI+ and ESI− modes after running a set of serum QC samples. The method we have developed enables the detection of a large number of metabolic features, of which around 70% can be attributed to sample-related compounds (after background subtraction and exclusion, removal of compounds not found in > 80% of QC samples, and exclusion of compounds with a QC CV > 30%, see supplementary information 2). A molecular formula could be attributed to around 80% of these metabolic features. Over 80% of these sample compounds had a QC CV < 15% demonstrating a good level of reproducibility across injections. As can be seen in Supplementary Fig. 2, peak areas of detected spiked internal standards displayed high reproducibility (< 10% RSD) and excellent mass accuracy (< 1 ppm) across QC injections throughout the run.

To improve confidence in metabolite annotation we also performed data dependent MS/MS across a range of masses in a similar way to (Mullard et al. 2015). As can be seen in Table 3 around 70% of sample compounds had associated MS/MS spectra with a large proportion of MS2 spectra corresponding to a preferred adduct ion ([M+ H]+).

As is commonly the case in untargeted metabolomics, the level of annotation and identification confidence was quite varied. In our analyses, annotation and identification ranged from identification levels 5-2 (Schymanski et al. 2014) (Table 3). We do find a small number of level 1 identifications in our study; however, our in-house mass and spectral libraries are small and still under development. For this reason, level 1 identifications will not be discussed. Results in terms of annotation and identification at various levels from both ESI+ and ESI− were comparable (Table 2); we will describe these for ESI+.

In ESI+ 3016 sample compounds had a full match to a proposed molecular formula (level 4). Of these, 1156 matched mass libraries in ChemSpider (level 3) on our searches against BioCyc, HMDB, KEGG, MassBank and NIST. In addition, around 700 compounds were matched against mass libraries provided by CD3.1 software along with our own imported ones (including the serum metabolome database (Psychogios et al. 2011) and the COCONUT Natural Products database (Sorokina and Steinbeck 2020)). Of the 2612 metabolic features with MS/MS spectra, 391 were matched with reasonable confidence (≥ 70%) to the mzCloud spectral library. Of these, 211 were fully matched against a proposed molecular formula by CD3.1 providing level 2 identification confidence. These are provided in Supplementary Information 3. These compounds represented diverse metabolite classes such as amino acids, peptides and analogues, lipids, and lipid-like molecules (including fatty acyls and steroids), as well as carboxylic acids and derivatives.

Of the remaining 165 compounds with ≥ 70% match to the mzCloud spectral library but without full match to a proposed molecular formula, a large proportion are not marked as having a full match to proposed molecular formulae for several reasons. This may include better spectral matching with other compounds sharing similar substructures, but with this providing a mass error > 3 ppm. In some cases, closer manual inspection is required whereas in others, the spectral matching provides clues as to the possible underlying substructure of the molecule in question. There is some level of duplication in annotation and putative identifications, either through close matches to mass or spectral libraries, or due to the same precursor mass appearing at more than one retention time (yet with different peak areas and intensities). Some of these can be seen in Supplementary Information 3, where 57 of 211 annotated compounds were duplicated in this way. As an example, we found Arachidonic acid eluting at 4 different retention times yet spectral matching of all these with mzCloud was > 90% in confidence. Another such example was Ecgonine, eluting at two different retention times yet both having excellent spectral matching to mzCloud (> 90% match). Further investigation may reveal this to be due to (positional) isomers and/or some compounds simply binding to the column and eluting over different times. This is of lesser importance for present purposes where we aim to find unique markers of transporter substrates.

Taking into account that these results are illustrative of a QC run, results in Table 3 suggests our LC-MS/MS method is a clear improvement on those shown in Table 1. The number of metabolic features after correction and exclusion in our study are lower than those in Ganna et al. (Ganna et al. 2015), however, the number of level 2 identified compounds (whether using (Sumner et al. 2007) or (Schymanski et al. 2014)) is nearly double. Whilst this is not the case when we compare against the number of level 2 identified compounds in (Dunn et al. 2015), we expect this will increase in our methodology as number of compounds and spectra in mzCloud grow, we match to spectral libraries outside of Compound Discoverer and we continue to increase our in-house library. Furthermore, our elution gradient is shorter (15 min in both ESI+ and ESI− vs. 22 min ES+ and 24 min ESI-) which will provide great time and cost savings.

**Table 3:**
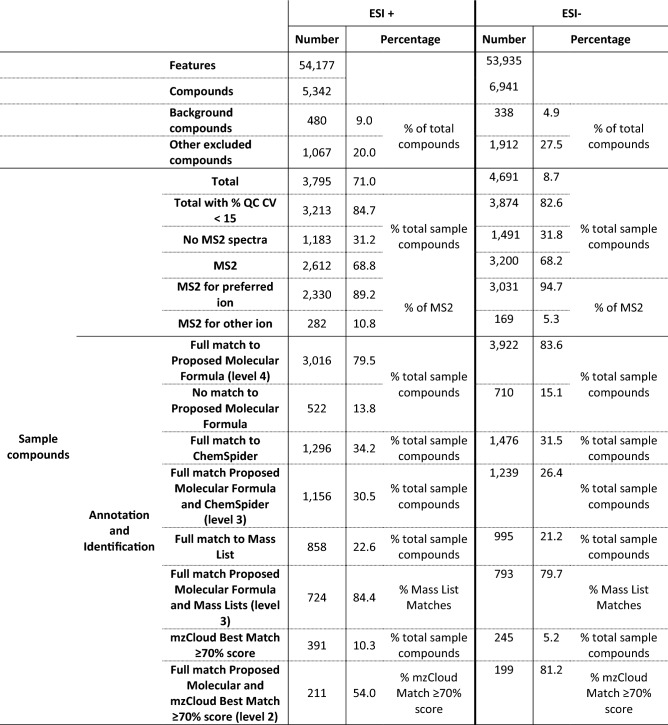
Summary of LC-MS/MS results of serum QC samples obtained following preprocessing using CD3.1

Note features here correspond to detected ions, compounds here correspond to what is commonly referred to as metabolic features (m/z and RT)

### Application of our method to determine the consumption and excretion of serum compounds by mammalian cell lines

One of the main purposes for developing the above untargeted LC-MS/MS methodology is to apply this to measure the uptake and excretion of serum compounds by mammalian cell lines. As a proof of principle, we assessed this by analysis in ESI+ of spent serum samples from 4 cell lines (A549, K562, SAOS2 and U2OS) incubated in serum at two different densities (2 and 4 million) at two timepoints (0 and 20 minutes incubation).

A summary table of the number of compounds detected and identified with various levels of confidence can be found in Supplementary Table S1. The results obtained are comparable to those shown in Table 1, demonstrating good reproducibility of our method.

**Fig. 2:**
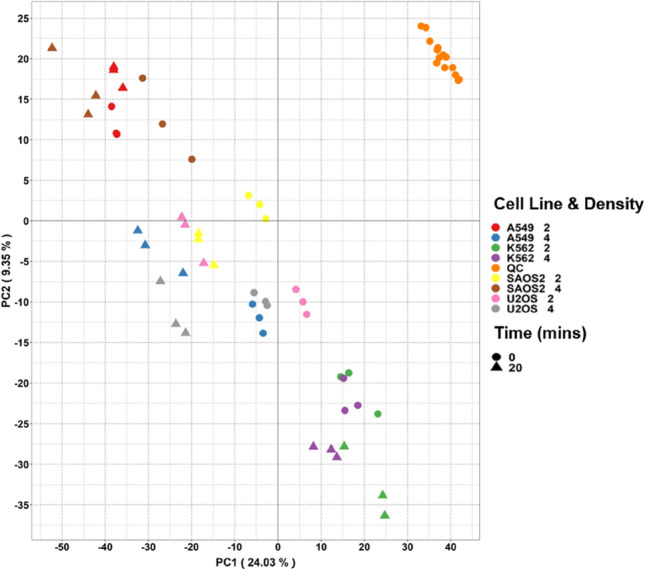
PCA scores plots of spent serum extracts following incubation for 0 or 20 minutes with 4 different cell lines and two densities. Colour: Cell line and density, shape: incubation time (Color figure online)

PCA reveals differences in samples (Fig. 2): the different cell lines fell into distinct groups separated in both PC1 and PC2. Furthermore, separations between time 0 and 20 min of incubation suggested differences in metabolic profile of spent serum influenced by time.

**Fig. 3:**
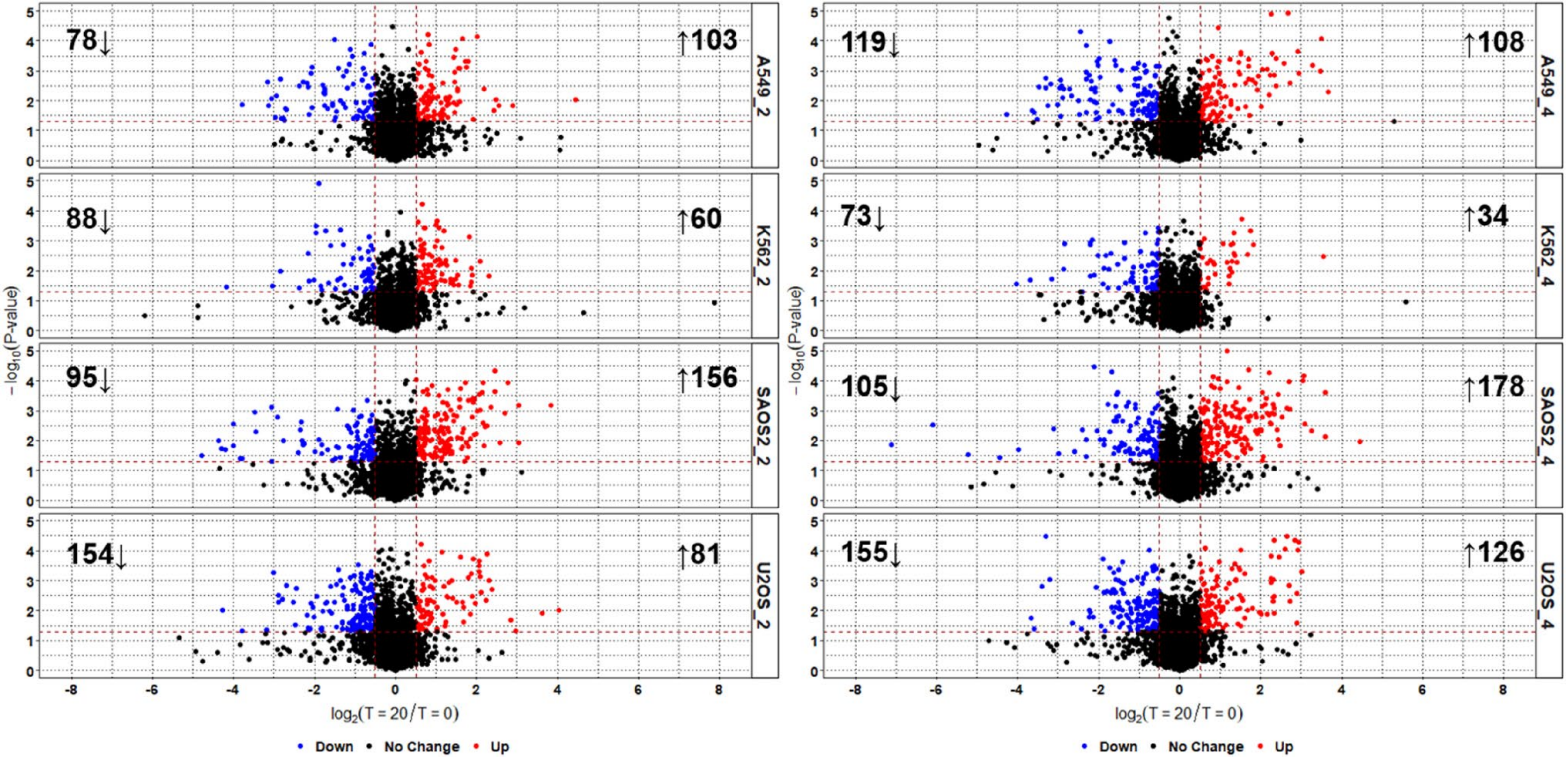
Volcano plots showing differences in number and magnitude of serum compound consumption and excretion by different cell lines and densities over 2 min. Threshold for significant change: P-value < 0.05 and log_2_ Fold Change < − 0.5 or > 0.5. Left panel: 2 million cell density; right side 4 million

To confirm that the separation of cell lines as well as the effect of incubation time and cell density are the result of differences in the uptake and excretion of serum components, we performed simple univariate analyses. The volcano plots in Fig. 3 confirm this is the case; cell lines consumed and excreted serum components however, the number of compounds consumed or excreted was not always proportional to increasing density, indicating that a rich set of metabolic activities were taking place during this period.

**Fig. 4:**
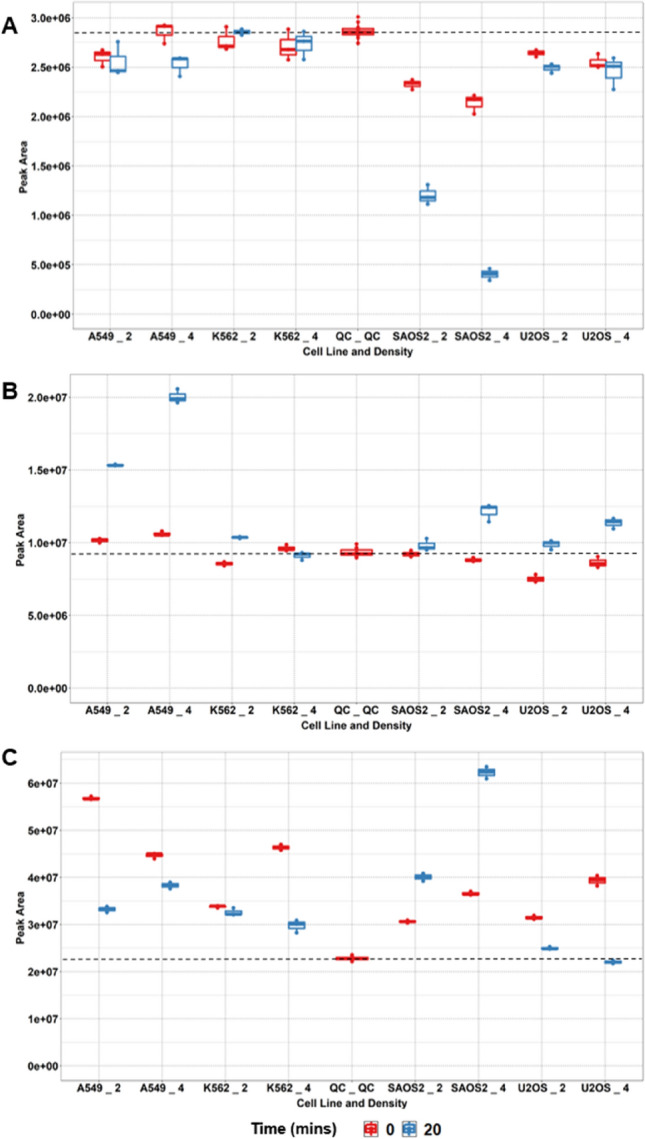
Cell- line specific consumed or secreted compounds **a** Unidentified compound with assigned molecular formula C21H37N5O7 consumed by SAOS2 cell lines only, **b** γ-L-Glutamyl-L-glutamic acid secreted exclusively by A549 cell lines. **c** Nicotinamide secreted by SAOS2 cell lines but consumed by others. X-axis labels: A549_2, A549 cells at 2 million density; A549_4, A549 cells at 4 million density; K562_2, K562 cells at 2 million density; A549_4, K562 cells at 4 million density; QC_QC, Quality Control; SAOS2_2, SAOS2 cells at 2 million density; SAOS2_4, SAOS2 cells at 4 million density; U2OS _2, U2OS cells at 2 million density; U2OS_4, U2OS cells at 4 million density. Horizontal dashed line added at the median level in QC sample to aid visualisation

Fuller results will be reported elsewhere for a much larger panel of cell lines; however, we provide examples of a compound exclusively consumed by one cell line (SAOS2, Fig. 4a), another exclusively secreted by another cell line (A549, Fig. 4b) and finally one where a mix of consumption and secretion was observed (Fig. 4c).

In SAOS2 cells, an unknown compound with mass of 471.26882 matching the molecular formula C21H37N507 was consumed exclusively by this cell lines, with consumption increasing in relation to higher cell density (Fig. 4a, log_2_ fold change − 0.84, P = 0.0004 at 2 million density and log2 fold change − 1.83, P = 0.0002 at 4 million density). Fragmentation data matching against mzCloud suggest some possible substructures of this compound match fragments for an environmental compound on the NORMAN suspect list (Mistrik et al. 2019) as shown in Supplementary Fig. 3A.

As can be seen in Fig. 4b, A549 cells secreted γ-L-Glutamyl-L-glutamic acid whilst the other 3 cell lines did not. The increase in levels of this metabolite was nearly double when cell density was doubled (log_2_ fold change 0.60, P = 0.0004 at 2 million density and log2 fold change 0.93, P = 0.0002 at 4 million density) whereas in other cell lines the changes were below our threshold (log_2_ fold change > 0.5 and P < 0.05). The identification of this compound is at a reasonable level 2, with 90.2% match to this compound in mzCloud, and low mass error (− 0.00029 Da or − 1.06 ppm) as can be seen in Supplementary Fig. 3B.

Nicotinamide (level 2 identification as shown in Supplementary Fig. 3C) was found to be secreted by SAOS2 cell lines in a density dependent manner (log_2_ fold change 0.55, P = 0.0010 at 2 million density and log2 fold change 0.98, P = 0.002 at 4 million density) yet consumed by K562 (log_2_ fold change − 0.89, P = 0.0050) and U2OS (log_2_ fold change − 1.26, P = 0.0004) cell lines at 4 million density and A549 cells at 2 million density only (log_2_ fold change of − 1.01, P = 0.0002).

While the biological significance of the consumed or secreted compounds in Figs. 3 and 4 will be discussed elsewhere in due course, the main message from this study is that the methodology employed here is robust and reproducible, (ii) capable of measuring the transport behaviour of serum compounds in an entirely unbiased way, (iii) and shows the massive differences between individual cell lines (O’Hagan et al. 2018; Wright Muelas et al. 2019).

## Discussion

We have here described an untargeted LC-MS/MS method developed to maximise the number and diversity of compounds detected in human serum whilst also acquiring sufficient fragmentation data for improved metabolite annotation confidence, all within a reasonable period of 15 minutes. The method enables detection of around 4000–5000 sample-related metabolic features in both ESI+ and ESI−, with excellent reproducibility and mass accuracy; across QC injections, ≥ 80% of sample compounds QC CVs were ≤ 15% (Table 3), and spiked internal standard QC CVs were < 10% (Supplementary Figure S2) with excellent mass accuracy (< 1 ppm).

Annotation and identification of metabolites is by far the greatest bottleneck encountered in untargeted metabolomics (Djoumbou Feunang et al. 2016; Dunn et al. 2013; Misra and van der Hooft 2016). Despite an increasing availability of mass spectral libraries (Vinaixa et al. 2016), only a small proportion of small molecules in these are derived from experimental data using pure standards and, even then, these seem to cover only around 40% of compounds within human genome scale metabolic network reconstructions (Frainay et al. 2018). Note that most serum metabolites have an exogenous source(O’Hagan and Kell 2017). Only a limited number of untargeted metabolomics studies of human serum using LC-MS/MS have sufficient details with which to compare our results (Table 1). Our method enables annotation of a significant number of metabolites at levels 4-2 (Table 3). From these, we found 226 metabolites with level 2 identification confidence in ESI+, representing diverse (and relevant) metabolite classes. The number of level 2 identified compounds using our method is also improved in comparison to results reported by Ganna et al. (Ganna et al. 2015). This is not the case when we compared (Dunn et al. 2015), however, the elution gradient in our method is shorter (15 min in both ESI+ and ESI− vs. 22 min ES+ and 24 min ESI−) which will provide great time and cost savings. The annotation and identification of metabolites to level 2 in our study is likely also limited using spectral libraries available through Compound Discoverer, namely mzCloud and local spectral libraries provided as standard with this software within mzVault. Another limitation within our reported data is the small number of level 1 metabolite identifications in our study; our in-house mass and spectral libraries are small and still under development.

In addition to maximising metabolite detection and identification, we have demonstrated the applicability of the LC-MS/MS method described to measure differences in the uptake and secretion of compounds by cell lines following incubation in human serum. This takes inspiration from work by Gründemann and colleagues (Gründemann et al. 2005) taking advantage of the complex mixture of candidate transporter substrates in human serum. The results reveal both the reproducibility of the analyses and distinct metabolic footprints(Allen et al. 2003) of different cell lines in terms of both uptake and secretion (Figs. 2 and 3).

As shown in Fig. 4, some compounds were consumed exclusively by certain cell lines and not others, whilst others were consumed by some but secreted by others. These differences are undoubtedly related to the transporter expression profiles of these cell lines. We have recently shown transporter expression to vary widely between cells and tissues which can be explained by the requirements of different tissues and cell lines for different amounts of specific substrates (O’Hagan et al. 2018). Fuller and more extensive results using a larger panel of different cell lines and time points will be reported elsewhere, and use of transcriptomic and proteomic transporter expression profiles to relate these to the potential substrates of transporter proteins.

## Conclusions

We have developed a new, 15-min untargeted metabolomics method using LC-MS/MS that allows for the robust and convenient measurement of a large number of metabolites in human serum. The method additionally acquires fragmentation data to enable improved annotation and identification of compounds. We also describe a protocol for investigating the natural substrates of transporters by way of incubating human cell lines in serum and using the above LC-MS/MS method to measure reproducible and unbiased differences in the uptake of serum compounds.

## Electronic supplementary material

Below is the link to the electronic supplementary material.

Supplementary file1 (DOCX 525 kb)

Supplementary file2 (XLSX 203 kb)

Supplementary file3 (XLSX 118 kb)

## Data Availability

The datasets generated during and/or analysed during the current study are not publicly available but are available from the corresponding author on reasonable request.

## References

[CR1] Allen J, Davey HM, Broadhurst D, Heald JK, Rowland JJ, Oliver SG, Kell DB (2003). High-throughput classification of yeast mutants for functional genomics using metabolic footprinting. Nature Biotechnology.

[CR2] Begley P, Francis-McIntyre S, Dunn WB, Broadhurst DI, Halsall A, Tseng A, Knowles J, Goodacre R, Kell DB (2009). Development and performance of a gas chromatography—Time-of-flight mass spectrometry analysis for large-scale nontargeted metabolomic studies of human serum. Analytical Chemistry.

[CR3] Borodina I, Kenny LC, McCarthy CM, Paramasivan K, Pretorius E, Roberts TJ, van der Hoek SA, Kell DB (2020). The biology of ergothioneine, an antioxidant nutraceutical. Nutrition Research Reviews.

[CR4] Broadhurst D, Goodacre R, Reinke SN, Kuligowski J, Wilson ID, Lewis MR, Dunn WB (2018). Guidelines and considerations for the use of system suitability and quality control samples in mass spectrometry assays applied in untargeted clinical metabolomic studies. Metabolomics.

[CR5] Broadhurst DI, Kell DB (2006). Statistical strategies for avoiding false discoveries in metabolomics and related experiments. Metabolomics.

[CR6] Brown M, Dunn WB, Dobson P, Patel Y, Winder CL, Francis-McIntyre S, Begley P, Carroll K, Broadhurst D, Tseng A, Swainston N, Spasic I, Goodacre R, Kell DB (2009). Mass spectrometry tools and metabolite-specific databases for molecular identification in metabolomics. The Analyst.

[CR7] Brown M, Dunn WB, Ellis DI, Goodacre R, Handl J, Knowles JD, O’Hagan S, Spasić I, Kell DB (2005). A metabolome pipeline: From concept to data to knowledge. Metabolomics.

[CR8] Cesar-Razquin A, Girardi E, Yang M, Brehme M, Saez-Rodriguez J, Superti-Furga G (2018). In silico prioritization of transporter-drug relationships from drug sensitivity screens. Frontiers in Pharmacology.

[CR9] Cesar-Razquin A, Snijder B, Frappier-Brinton T, Isserlin R, Gyimesi G, Bai X, Reithmeier RA, Hepworth D, Hediger MA, Edwards AM, Superti-Furga G (2015). A call for systematic research on solute carriers. Cell.

[CR10] Cho K, Mahieu NG, Johnson SL, Patti GJ (2014). After the feature presentation: Technologies bridging untargeted metabolomics and biology. Current Opinion in Biotechnology.

[CR11] Djoumbou Feunang Y, Eisner R, Knox C, Chepelev L, Hastings J, Owen G, Fahy E, Steinbeck C, Subramanian S, Bolton E, Greiner R, Wishart DS (2016). ClassyFire: Automated chemical classification with a comprehensive, computable taxonomy. Journal of Cheminformatics.

[CR12] Dobson PD, Kell DB (2008). Carrier-mediated cellular uptake of pharmaceutical drugs: An exception or the rule?. Nat Rev Drug Discov.

[CR13] Dunn WB, Broadhurst D, Begley P, Zelena E, Francis-McIntyre S, Anderson N, Brown M, Knowles JD, Halsall A, Haselden JN, Nicholls AW, Wilson ID, Kell DB, Goodacre R, Serum H, Metabolome C (2011). Procedures for large-scale metabolic profiling of serum and plasma using gas chromatography and liquid chromatography coupled to mass spectrometry. Nature Protocals.

[CR14] Dunn WB, Broadhurst DI, Deepak SM, Buch MH, McDowell G, Spasic I, Ellis DI, Brooks N, Kell DB, Neyses L (2007). Serum metabolomics reveals many novel metabolic markers of heart failure, including pseudouridine and 2-oxoglutarate. Metabolomics.

[CR15] Dunn WB, Erban A, Weber RJM, Creek DJ, Brown M, Breitling R, Hankemeier T, Goodacre R, Neumann S, Kopka J, Viant MR (2013). Mass appeal: Metabolite identification in mass spectrometry-focused untargeted metabolomics. Metabolomics.

[CR16] Dunn WB, Lin W, Broadhurst D, Begley P, Brown M, Zelena E, Vaughan AA, Halsall A, Harding N, Knowles JD, Francis-McIntyre S, Tseng A, Ellis DI, O’Hagan S, Aarons G, Benjamin B, Chew-Graham S, Moseley C, Potter P, Winder CL, Potts C, Thornton P, McWhirter C, Zubair M, Pan M, Burns A, Cruickshank JK, Jayson GC, Purandare N, Wu FCW, Finn JD, Haselden JN, Nicholls AW, Wilson ID, Goodacre R, Kell DB (2015). Molecular phenotyping of a UK population: Defining the human serum metabolome. Metabolomics.

[CR17] Dunn WB, Wilson ID, Nicholls AW, Broadhurst D (2012). The importance of experimental design and QC samples in large-scale and MS-driven untargeted metabolomic studies of humans. Bioanalysis.

[CR18] Frainay C, Schymanski EL, Neumann S, Merlet B, Salek RM, Jourdan F, Yanes O (2018). Mind the gap: Mapping mass spectral databases in genome-scale metabolic networks reveals poorly covered areas. Metabolites.

[CR19] Ganna A, Fall T, Salihovic S, Lee W, Broeckling CD, Kumar J, Hägg S, Stenemo M, Magnusson PKE, Prenni JE, Lind L, Pawitan Y, Ingelsson E (2015). Large-scale non-targeted metabolomic profiling in three human population-based studies. Metabolomics.

[CR20] Garg N, Kapono CA, Lim YW, Koyama N, Vermeij MJA, Conrad D, Rohwer F, Dorrestein PC (2015). Mass spectral similarity for untargeted metabolomics data analysis of complex mixtures. International Journal of Mass Spectrometry.

[CR21] Ghatak S, King ZA, Sastry A, Palsson BO (2019). The y-ome defines the 35% of *Escherichia coli* genes that lack experimental evidence of function. Nucleic Acids Research.

[CR22] Girardi E, César-Razquin A, Lindinger S, Papakostas K, Konecka J, Hemmerich J, Kickinger S, Kartnig F, Gürtl B, Klavins K, Sedlyarov V, Ingles-Prieto A, Fiume G, Koren A, Lardeau C-H, Kandasamy K, Kubicek R, Ecker S, Superti-Furga G (2020). A widespread role for SLC transmembrane transporters in resistance to cytotoxic drugs. Nature Chemical Biology.

[CR23] Gründemann D (2012). The ergothioneine transporter controls and indicates ergothioneine activity—A review. Preventive Medicine.

[CR24] Gründemann D, Harlfinger S, Golz S, Geerts A, Lazar A, Berkels R, Jung N, Rubbert A, Schömig E (2005). Discovery of the ergothioneine transporter. Proceedings of the National Academy of Sciences USA.

[CR25] Hediger MA, Romero MF, Peng JB, Rolfs A, Takanaga H, Bruford EA (2004). The ABCs of solute carriers: Physiological, pathological and therapeutic implications of human membrane transport proteinsIntroduction. Pflugers Archiv.

[CR26] Jiang M, Chen T, Feng H, Zhang Y, Li L, Zhao A, Niu X, Liang F, Wang M, Zhan J, Lu C, He X, Xiao L, Jia W, Lu A (2013). Serum metabolic signatures of four types of human arthritis. Journal of Proteome Research.

[CR27] Jindal S, Yang L, Day PJ, Kell DB (2019). Involvement of multiple influx and efflux transporters in the accumulation of cationic fluorescent dyes by *Escherichia coli*. BMC Microbiology.

[CR28] Kell DB (2020). Hitchhiking into the cell. Nature Chemical Biology.

[CR29] Kell DB, Dobson PD, Bilsland E, Oliver SG (2013). The promiscuous binding of pharmaceutical drugs and their transporter-mediated uptake into cells: What we (need to) know and how we can do so. Drug Discovery Today.

[CR30] Kell DB, Dobson PD, Oliver SG (2011). Pharmaceutical drug transport: The issues and the implications that it is essentially carrier-mediated only. Drug Discovery Today.

[CR31] Kell DB, Oliver SG (2014). How drugs get into cells: Tested and testable predictions to help discriminate between transporter-mediated uptake and lipoidal bilayer diffusion. Frontiers in Pharmacology.

[CR32] Kell DB, Wright Muelas M, O'Hagan S, Day PJ (2018). The role of drug transporters in phenotypic screening. Drug Target Review.

[CR33] Kenny LC, Broadhurst DI, Dunn W, Brown M, North RA, McCowan L, Roberts C, Cooper GJS, Kell DB, Baker PN (2010). Robust early pregnancy prediction of later preeclampsia using metabolomic biomarkers. Hypertension.

[CR34] Lex A, Gehlenborg N, Strobelt H, Vuillemot R, Pfister H (2014). UpSet: Visualization of intersecting sets. IEEE Transactions on Visualization and Computer Graphics.

[CR35] Martin J-C, Maillot M, Mazerolles G, Verdu A, Lyan B, Migné C, Defoort C, Canlet C, Junot C, Guillou C, Manach C, Jabob D, Bouveresse D J-R, Paris E, Pujos-Guillot E, Jourdan F, Giacomoni F, Courant F, Favé G, Le Gall G, Chassaigne H, Tabet J-C, Martin J-F, Antignac J-P, Shintu L, Defernez M, Philo M, Alexandre-Gouaubau M-C, Amiot-Carlin M-J, Bossis M, Triba MN, Stojilkovic N, Banzet N, Molinié R, Bott R, Goulitquer S, Caldarelli S, Rutledge DN (2015). Can we trust untargeted metabolomics? Results of the metabo-ring initiative, a large-scale, multi-instrument inter-laboratory study. Metabolomics.

[CR36] Misra BB, van der Hooft JJJ (2016). Updates in metabolomics tools and resources: 2014–2015. Electrophoresis.

[CR37] Mistrik, R., Aligizakis, N., Schymanski, E., & Williams, A. (2019) S19 | MZCLOUD | mzCloud Compounds (Version NORMAN-SLE-S19.0.2.0).

[CR38] Mullard G, Allwood JW, Weber R, Brown M, Begley P, Hollywood KA, Jones M, Unwin RD, Bishop PN, Cooper GJS, Dunn WB (2015). A new strategy for MS/MS data acquisition applying multiple data dependent experiments on Orbitrap mass spectrometers in non-targeted metabolomic applications. Metabolomics.

[CR39] O’Hagan S, Dunn WB, Brown M, Knowles JD, Kell DB (2005). Closed-loop, multiobjective optimization of analytical instrumentation: Gas chromatography/time-of-flight mass spectrometry of the metabolomes of human serum and of yeast fermentations. Analytical Chemistry.

[CR40] O’Hagan S, Kell DB (2017). Consensus rank orderings of molecular fingerprints illustrate the ‘most genuine’ similarities between marketed drugs and small endogenous human metabolites, but highlight exogenous natural products as the most important ‘natural’ drug transporter substrates. ADMET & DMPK.

[CR41] O’Hagan S, Wright Muelas M, Day PJ, Lundberg E, Kell DB (2018). GeneGini: Assessment via the gini coefficient of reference “housekeeping” genes and diverse human transporter expression profiles. Cell Syst.

[CR42] Psychogios N, Hau DD, Peng J, Guo AC, Mandal R, Bouatra S, Sinelnikov I, Krishnamurthy R, Eisner R, Gautam B, Young N, Xia J, Knox C, Dong E, Huang P, Hollander Z, Pedersen TL, Smith SR, Bamforth F, Greiner R, McManus B, Newman JW, Goodfriend T, Wishart DS (2011). The human serum metabolome. PLoS One.

[CR43] Schymanski EL, Jeon J, Gulde R, Fenner K, Ruff M, Singer HP, Hollender J (2014). Identifying small molecules via high resolution mass spectrometry: Communicating confidence. Environmental Science & Technology.

[CR100] Sorokina, M., & Steinbeck, C. (2020). Review on natural products databases: Where to find data in 2020. *Journal of Cheminformatics*, *12*(1), 20. 10.1186/s13321-020-00424-9 PMC7118820 33431011

[CR44] Sumner LW, Amberg A, Barrett D, Beale MH, Beger R, Daykin CA, Fan TWM, Fiehn O, Goodacre R, Griffin JL, Hankemeier T, Hardy N, Harnly J, Higashi R, Kopka J, Lane AN, Lindon JC, Marriott P, Nicholls AW, Reily MD, Thaden JJ, Viant MR (2007). Proposed minimum reporting standards for chemical analysis. Metabolomics.

[CR45] Superti-Furga G, Lackner D, Wiedmer T, Ingles-Prieto A, Barbosa B, Girardi E (2020). The RESOLUTE consortium: Unlocking SLC transporters for drug discovery. Nature Review Drug Discovery.

[CR46] Tautenhahn R, Cho K, Uritboonthai W, Zhu Z, Patti GJ, Siuzdak G (2012). An accelerated workflow for untargeted metabolomics using the METLIN database. Nature Biotechnology.

[CR47] Treutler H, Tsugawa H, Porzel A, Gorzolka K, Tissier A, Neumann S, Balcke GU (2016). Discovering regulated metabolite families in untargeted metabolomics studies. Analytical Chemistry.

[CR48] Vaidyanathan S, Broadhurst DI, Kell DB, Goodacre R (2003). Explanatory optimization of protein mass spectrometry via genetic search. Analytical Chemistry.

[CR49] Vinaixa M, Schymanski EL, Neumann S, Navarro M, Salek RM, Yanes O (2016). Mass spectral databases for LC/MS- and GC/MS-based metabolomics: State of the field and future prospects. TrAC Trends in Analytical Chemistry.

[CR50] Wright Muelas M, Mughal F, O’Hagan S, Day PJ, Kell DB (2019). The role and robustness of the Gini coefficient as an unbiased tool for the selection of Gini genes for normalising expression profiling data. Scientific Reports.

[CR51] Zelena E, Dunn WB, Broadhurst D, Francis-McIntyre S, Carroll KM, Begley P, O’Hagan S, Knowles JD, Halsall A, Consortium H, Wilson ID, Kell DB (2009). Development of a robust and repeatable UPLC-MS method for the long-term metabolomic study of human serum. Analytical Chemistry.

